# Determinates of depressive disorder among adult patients with cardiovascular disease at outpatient cardiac clinic Jimma University Teaching Hospital, South West Ethiopia: cross-sectional study

**DOI:** 10.1186/s13033-019-0269-8

**Published:** 2019-03-05

**Authors:** Halima Umer, Alemayehu Negash, Mengesha Birkie, Asmare Belete

**Affiliations:** 1 Research and Training Department of Amanuel Mental Specialized Hospital, Addis Abeba, Ethiopia; 2 Department of Psychiatry, College of Public Health and Medical Sciences, Jimma University, Jimma, Ethiopia; 3 Department of Psychiatry, College of Medicine and Health Sciences, Wollo University, Dessie, Ethiopia

**Keywords:** Depression, Cardiovascular disease, Ethiopia, Determinants, Patient health questionnaire-9

## Abstract

**Background:**

Depression and heart disease are an important public-health problem. Depression is one of the most prevalent and disabling psychiatric disorders with more than three times increased risk among patients with cardiovascular disorders.

**Objective:**

To identify the prevalence and associated factors of depressive disorder among adult patients with cardiovascular disease.

**Methods:**

Institution based cross-sectional study design was used to conduct this study on 293 study participants attending an outpatient cardiac clinic at Jimma University Teaching Hospital. All eligible patients were recruited into the study consecutively. Depression was assessed using patient health questionnaire-9. The patient health questionnaire-9 had a total score of 27, from which 0–4: no/minimal depression, 5–9: mild depression, 10–14: moderately depression, 15–19: moderately severe depression and 20–27 severe depression. The data was feed into Epi-data version 3.1 and lastly exported to SPSS version 21 for analysis. Bivariate analysis was used to analyze the statistical association of covariates of interest with depressive disorder among patients with cardiovascular disease. Then, logistic regression analysis was used as a final model to control confounders. The strength of association was measured by a 95% confidence interval.

**Results:**

A total of 293 adult patients diagnosed with the cardiovascular disease were included in the study with 97% (n = 284) of response rate, 47.2% (n = 134) males and 52.8% (n = 150) females, making female to a male ratio around 1.1:1. The prevalence of depression was 52.8% (n = 150/284). Out of the subjects with depression 52.67% (n = 79), 36.0% (n = 54) and 11.33% (n = 17) were mild, moderate and severe depression, respectively. Variables such as employed, unemployed, physical activity, current cigarette user and poor social support were independently associated with depression in the final model.

**Conclusions:**

In this study, depression was found to be highly prevalent psychiatric comorbidity in adult cardiovascular disease patients.

## Background

Non-communicable diseases (NCD) currently account 60% of all deaths and 48% of the disability-adjusted life years (DALYs) worldwide, 40% for communicable diseases. In 2008, four out of five NCD deaths occurred in low- and middle-income countries [1]. Mental disorders are a major contributor to the burden of disease in all regions of the world [2]. Mental health conditions are the leading cause of DALYs worldwide and account for 37% of years of life lost (YLL) from NCDs [1].

According to the World Health Organization (WHO) report in 2011 unipolar depressive disorder is the third leading cause of disease burden worldwide. Mental disorders account for 25.3% and 33.5% of all years lived with a disability in low- and middle-income countries (LAMICs), respectively [3]. Mental illness is both a direct cause of mortality and a major risk factor for adverse health outcomes [4].

Depression is a substantial contributor to the global burden of disease. It affects people in all communities worldwide. In 2012, the WHO reported that depression is estimated to affect 350 million peoples [5]. Depression is a common mental disorder that has a clinical feature of depressed mood, loss of interest, reduced energy, feelings of guilt or low self-esteem, disturbance of sleep/appetite, and poor concentration, severe enough to cause severe impairment in important role function. At its worst, depression leads to suicide and is responsible for 1 million deaths due to suicide every year, which translates to 3000 suicide deaths every day [5].

World Health O report shows that 17.3 million people died from CVDs, contributing to 30% of all global deaths and this represents 50% of all death from NCD. In the same year, CVDs were the number one cause of death and deaths from these disorders in LAMIC constituted 80% [6, 7]. At the same time, it is estimated that about 19 million deaths occur annually from cardiovascular causes in lower-income countries [1, 6]. Mental health and cardiovascular diseases account for almost 70% of global economic losses [1].

The reported prevalence of major depression in patients with CAD is around 20%, the prevalence of depression is among patient with CHD is very high (31–34%) and is about three times more compared to that in the general population. Moreover, depression has been found to be a risk factor for the development of CAD and worsens its outcome when depression co-exists with established CAD [19–21]. One-fifth of the patients with CHD attending outpatients and one-third of patients with congestive heart failure (CHF) have co-existing depression.

Depression in patients with CHD and CHF, however, is not recognized or not properly treated most of the time [8].

Quality of life of patients and their significant others are seriously affected by debilitating physical health, psychological distress, and diminished family role [9]. In patients undergoing coronary artery bypass graft (CABG), depression has been associated with longer hospitalization, poorer functional outcomes, more preoperative complications, higher rates of mortality and more symptomatic before and also after surgery [10, 11].

Effects of untreated depression that include: increased risk of a coronary event, decreased feelings of wellbeing and quality of life (QOL), decreased medication compliance and decreased risk factor modification. Can seriously increase the risks of further cardiac morbidity or mortality. Despite this knowledge; Physicians usually under-detect depression among cardiac patients; moreover, some of the cardiovascular drugs such as beta blockers may worsen the overall depressive symptoms [12, 13].

Factors associated with depression, most studies have found that younger patients and women were more likely to have the disorder in the context of CVD [16]. Poor social support, prior ACS, and in some cases, comorbid diabetes may also increase depression risk [14–16].

Many studies implicate depression among CAD patients to have the association with female sex, younger age, living situation, prior ACS, comorbid diabetes, HF severity class, low educational attainment, use of beta blockers, being housewives, unemployment status [14, 17–21]. Positive family history of depression, smoking, history of CHD/MI, social isolation/social support, NYHA CLASS classification system based on clinical severity and prognosis, antihypertensive medication [22, 23]. People with depression are 25 to 40% more likely to die from heart disease than people without depression [24].

Furthermore, several candidate biological and behavioral factors believed to be mechanisms through which depression could lead to cardiac events have been identified. Such as tricyclic antidepressants toxicity associated with cardiac risk factors. Potential behavior related mechanisms comprise dietary factors, sedentary lifestyle with lack of exercise, non-adherence to cardiac prevention and treatment regimens, lack of optimal social support, unhealthy lifestyles such as smoking, alcoholism and reduced functional capacity [8, 25].

Therefore, the fact that depression is a predictor of cardiac events among patients with CVD is has gained universal consensus although such information is lacking from countries such as Ethiopia. Furthermore, it was a well-known fact that comorbid depression increases negative events such as progression to arteriosclerosis, increased health care utilization and increased hospital readmissions before or after a cardiac event.

In spite of, evidence has suggested that depression can aggravate the course of multiple cardiovascular conditions, detection of depression in cardiac patient remains low. According to the principal investigator’s knowledge, there is no study conducted in Ethiopia and the extent of the problem is not yet known. A better understanding of the extent of depression and associated factors is significant. This research will narrow this huge gap and will serve as a baseline for further researches to be undertaken on the subject matter.

## Methods and materials

### Study setting and period

The study was conducted in Jimma University Teaching Hospital (JUTH) outpatient cardiac clinic from October to November 2014. It has been running a public health care institution under the ministry of health by different names at a different time. Geographically, it is located in Jimma town 352 km south west of Addis Ababa. It provides service for at least 15 million populations residing in South-West Ethiopia. The cardiac clinic is one of the follow-up clinics giving service for a patient with chronic CVDs among other clinics that give service for patients with other chronic NCDs [26].

### Participants

The study participants were all adult patients who had CVD age 18 years and above who came for follow-up at JUTH cardiac clinic during the study period. A total of 293 adult patients who had CVD were involved in the study. This study exclude, individual who seriously ill and were not able to give information, had a severe neuropsychiatric condition or language deficit that would preclude informed consent or valid assessment was excluded.

### Measurements

The dependent variable was depression. The independent variables include socio-demographic characteristics such as age, sex, marital status, educational status, place of residence, income, empowerment, psychosocial, medication, clinical-related, behavioral and lifestyle factors.

### Data collection procedures and instruments

Data were collected using interview-administered questionnaires. Questionnaires were abstracted and adapted from different literature sources and modified according to the local context. The questionnaire prepared by the English language, translate to Afan Oromo and Amharic language by the language department who is a native speaker for oromifa language and back translation also made. Depression was measured using patient health questionnaire nine (PHQ-9) which is a validated instrument in Ethiopia [27]. The PHQ-9 has a total score of 27, from which 0–4: no/minimal depression, 5–9: mild depression, 10–14: moderately depression, 15–19: moderately severe depression and 20–27 severe depression. Morisky medical adherence scale-8 (MMAS-8) was used to assess medication adherence which is a validated tool in Ethiopia to screening and monitoring in clinical practice to identify and monitor high-risk non-adherent patients [28].

Oslo 3-items social support scale (OSS-3) was used to measure the strength of social support [29]. Pretest of the questionnaire was carried out on 5% of respondents; whose socio-demographic factors are the same with those actual study participants; from the cardiac clinic in Jimma University Teaching Hospital (JUTH) those individuals not included in the main study differentiated by making a mark on their chart.

### Data processing, analysis, interpretation, and presentation

The quantitative data were entered into the computer by using Epi-data version 3.1 and lastly exported to SPSS version 21 for analysis. Bivariate analysis and multiple logistic regressions were used. Finally, variables had a P-value of less than .25 on binary logistic regression were entered into multivariable logistic regression. The strength of association was measured by 95% confidence interval and Then P-value < .05 considered as significantly associated variables with depression in the final model. The result was presented by frequency tables, graphs and discussed with previous study findings.

### Ethical consideration

The ethical approval of the study was obtained from the Ethical Review Board of Jimma University, college of public health and Medical Sciences. Oral consent was obtained from the study participants. Patient those found to be severally depressed and suicidal risk appropriate intervention was done according to PHQ-9 score.

## Results

### Socio-demographic characteristics

A total of 293 adult patients diagnosed with CVD were included in the study with a response rate of 97% (n = 284). Out of the total respondents 47.2% (n = 134) males and 52.8% (n = 150) females with male-to-female ratio of 1.1:1. The age of the respondents ranged from 18 to 90 with a mean (SD) of 50.3 (17.13) years.

Nearly to two-third of (64.4%) patients came from the rural area and nearly three-fourths of the respondents were married (76.0%). About one-half of the total participants were illiterate (53.2%) (n = 151). Regarding the occupation status, more than one half of the total of the respondents were farmers, 50.7% (n = 144). Unemployed and housewives constituted 18% (n = 144) and 8.8% (n = 25) respectively. About 26.1% (n = 74) of the study participants claimed to earn an annual average income of 10,800 birrs (Table 1).

**Table 1:** Socio-demographic characteristics of the respondents in Jimma University Teaching Hospital cardiac clinic, 2014–2015 (n = 284)

Characteristics	Non depressed (PHQ-9 < 5) N (%)	Depressed (PHQ-9 ≥ 5) N (%)	Total (%)
Sex			
Male	60 (44.8)	74 (49.3)	134 (47.2)
Female	74 (55.2)	76 (50.7)	150 (52.8)
Age			
18–26	17 (12.7)	17 (11.3)	34 (12.0)
27–35	15 (11.2)	21 (14.0)	36 (12.7)
36–44	20 (14.9)	13 (8.7)	33 (11.6)
45–53	22 (16.4)	25 (16.7)	47 (16.5)
–62 ref	28 (20.9)	26 (17.3)	54 (19.0)
63–71	22 (16.4)	28 (18.7)	50 (17.6)
≥ 72	10 (7.5)	20 (13.3)	30 (11.2)
Marital status			
Single	20 (14.9)	13 (8.7)	33 (11.6)
Married	105 (78.4)	111 (74.0)	216 (76.0)
Divorced/separate	4 (3.0)	10 (6.7)	14 (5.0)
Widowed	5 (3.7)	16 (10.7)	21 (7.4)
Educational status			
Illiterate	67 (5.0)	84 (56.0)	151 (53.2)
Able to read and write only	20 (14.9)	36 (24.0)	56 (19.7)
Primary (1–8)	28 (20.9)	19 (12.7)	47 (16.5)
Secondary (9–12)	14 (10.4)	7 (4.7)	21 (7.4)
Tertiary (+12)	5 (3.7)	4 (2.7)	9 (3.2)
Occupational status			
Unemployed	16 (11.9)	35 (23.3)	51 (18.0)
Employed	12 (9.0)	4 (2.7)	16 (5.6)
Farmer	70 (52.2)	74 (49.3)	144 (50.7)
Merchant	9 (6.7)	10 (6.7)	19 (6.7)
Retired	7 (5.2)	5 (3.3)	12 (4.2)
Housewife	11 (8.2)	14 (9.3)	25 (8.8)
Other	9 (6.7)	8 (5.3)	17 (6.0)
Residence			
Rural areas	80 (59.7)	103 (68.7)	183 (64.4)
Urban areas	54 (40.3)	47 (31.3)	101 (35.6)
Average annual income			
0–999	33 (24.6)	37 (24.7)	70 (24.6)
1000–3599	32 (23.9)	37 (24.7)	69 (24.3)
3600–10,799	28 (20.9)	43 (28.7)	71 (25.0)
≥ 10,800	41 (30.6)	33 (22.0)	74 (26.1)

Other employment status—student, daily labor and house servants

### Psychosocial and behavioral factors

The majority (90.5%) of respondents were living with their family and 6.3% (n = 18) were living alone. Only 24.4% (n = 69) reported having good social support whereas 35.2% (n = 100) claimed to have poor social support. Those who reported having almost satisfactory or moderate social support constituted 40.5% (n = 115). About half of respondent (48.9%) do not do moderate to vigorous activities for 30 min or more, at least 4 days in a week. Respondents who used that in their lifetime were 26.8% (n = 76) and 8.8% (n = 25) were current use (Table 2).

**Table 2:** Psychosocial and behavioral related characteristics of the respondents in Jimma University Teaching Hospital cardiac clinic, 2014–2015 (n = 284)

Characteristics	Non depressed N (%)	Depressed N (%)	Total N (%)
Living condition			
Alone	6 (4.5)	12 (8.0)	18 (6.3)
With family	121 (90.3)	136 (90.7)	257 (90.5)
Other^a^	7 (5.2)	2 (1.3)	9 (3.2)
Oslo 3-items social support scale			
Poor support	38 (28.4)	62 (41.3)	100 (35.2)
Moderate support	62 (46.3)	53 (35.3)	115 (40.5)
Strong support	34 (25.4)	35 (23.3)	69 (24.3)
Physical activity			
Yes	79 (59.0)	66 (44.0)	145 (51.1)
No	55 (41.0)	84 (56.0)	139 (48.9)
Life time used substances			
Cigarette			
Yes	9 (6.7)	17 (11.3)	26 (9.2)
No	125 (93.3)	133 (88.7)	258 (90.8)
Alcohol			
Yes	19 (14.2)	15 (10.0)	34 (12.0)
No	115 (85.8)	135 (90.0)	250 (88.0)
Khat			
Yes	33 (24.6)	43 (28.7)	76 (26.8)
No	101 (75.4)	107 (71.3)	208 (73.2)
Cannabis			
Yes	4 (3.0)	6 (4.0)	10 (3.5)
No	130 (97.0)	144 (96.0)	274 (96.5)
Current used substances			
Cigarette			
Yes	3 (2.2)	11 (7.3)	14 (4.9)
No	131 (97.8)	139 (92.7)	270 (95.1)
Alcohol			
Yes	7 (5.2)	11 (7.3)	18 (6.3)
No	127 (94.8)	139 (92.7)	266 (93.7)
Khat			
Yes	14 (10.4)	11 (7.3)	25 (8.8)
No	120 (89.6)	139 (92.7)	259 (91.2)
Cannabis			
Yes	2 (1.5)	3 (2.0)	5 (1.8)
No	132 (98.5)	147 (98.0)	279 (98.2)

Living condition other^a^ With relative/friends/homeless

### Clinical characteristics and medication related factors

#### Medication related factors

The most prescribed drugs were diuretics in 75.0% (n = 213); angiotensin-converting enzyme inhibitors in 55.3% (n = 157) and beta-blockers in 45.2% (n = 137). The rate of depression was higher at 77.3% (n = 116) among participants who were taking diuretics. The majority of participants, 32.0% (n = 91) were ≤ 1 year of duration of treatment. The rate of depression is higher among those whose duration of treatment for CVD was greater than or equal to 5 years 30.7% (n = 46). Nearly three-fourth of participants (n = 206) were adherence to CVD medication (Table 3).

**Table 3:** Depression relation to medication taken by CVD patients, JUTH cardiac clinic, 2014–2015 (n = 284)

Characteristics	Total N (%)	Nondepressed N (%)	Depressed N (%)
Medication			
Digoxin	62 (21.8)	33 (24.6)	29 (19.3)
Diuretic	213 (75.0)	97 (72.4)	116 (77.3)
Beta blocker	137 (45.2)	72 (53.7)	65 (43.3)
Angiotensin-converting enzyme inhibitor	157 (55.3)	72 (53.7)	85 (56.7)
Calcium channel blocker	24 (8.5)	9 (6.7)	15 (10.0)
Anticoagulant	149 (52.5)	70 (52.2)	79 (52.7)
Statine (lipid lowering agents)	22 (7.7)	11 (8.2)	11 (7.3)
Penicillin	23 (8.1)	10 (7.5)	13 (8.7)
Other?	17 (6.0)	8 (6.0	9 (6.0)
Duration of treatment (year)			
≤ 1	91 (32.0)	50 (37.3)	41 (27.3)
1–2	51 (18.0)	24 (17.9)	27 (18.0)
2–3	29 (10.2)	12 (9.0)	17 (11.3)
3–4	30 (10.6)	11 (8.2)	19 (12.7)
≥ 5	83 (29.2)	37 (27.6)	46 (30.7)
Medication adherence			
Non adherent	78 (27.5)	45 (30.0)	33 (24.6)
Adherent	206 (72.5)	105 (70.0)	101 (75.4)

Other medications—vasodilator, broncho-dilator, steroid, PTU, insulin, metformine, daonil, valsartan

#### Clinical related factors

The majority of participants were diagnosed with Hypertensive heart disease (HHD) constituting 35.9% (n = 102). Cases with CHD were found in 26.4% (n = 75) whereas patients with Valvular heart disease (VHD) were found in 12.3% (n = 35). The highest rate of depression was found among HHD 36.0% (n = 54) and the lowest in other diagnostic groups of CVD (Corpulmonary, acute rheumatic fever, congenital heart disease, pericarditis and CHF due to thyrotoxicosis) classification 6.0% (n = 9). Among depressed 30.0% (n = 45) were the age of onset of CVD above 60 years. Close to one-third (30%) of the patients have been ill with CVD for the duration of 1–3 years.

Majority of participants 87.3% (n = 248) had no family history of mental illness. From those who had a family history of mental illness (55.6%; n = 20/36) were more depressed as compared to those no history of depression (44.4%; n = 16/36). Two hundred seventy (95.1%) of participants had no comorbid diabetes mellitus. Among those who had comorbid diabetes mellitus, most had depressed mood (64.3%; n = 9/14) as compared to that non-comorbid diabetes mellitus (35.7%; n = 5/14) with depression. Majority of participants who had comorbid diabetes mellitus 72.7% (n = 8) had onset ≤ 3 years after CVD illness. They had more depression (71.4%) as compared to those who had > 3 years of duration of comorbid DM after the onset of CVD illness.

Participants those who had comorbid hypertension constituted 45.1% (n = 128). Among CVD patients with comorbid hypertension, depression prevalence was 46.0% (n = 69). Among those who had comorbid hypertension their duration of onset ≤ 3 years, after CVD illness 64.0% (n = 33) were more depressed as compared to duration > 3 years of comorbid hypertension. Majority of the participants 60.9% (n = 173) with the previous history of hospitalization had more prevalence rate of depression (64.0%), as compared to previously none hospitalized for CVD patients (Table 4).

**Table 4:** Distribution of respondents by clinical related factors in JUTH cardiac clinic, 2014–2015 (n = 284)

Characteristics	Total N (%)	No depressed N %	Depressed N (%)
Diagnosis type of CVD illness			
Hypertensive heart diseases	102 (35.9)	48 (35.8)	54 (36.0)
Ischemic heart disease	75 (26.4)	37 (27.6)	38 (25.3)
Valvular heart disease	35 (12.3)	11 (8.2)	24 (16.0)
Rheumatic heart disease	26 (9.2)	14 (10.4)	12 (8.0)
Cardiomyopathy	29 (10.2)	16 (11.9)	13 (8.7)
Other	17 (5.0)	8 (6.0)	9 (6.0)
Duration of CVD illness (year)			
≤ 1	68 (23.9)	36 (26.9)	32 (21.3)
1–3	78 (27.5)	37 (27.6)	41 (27.3)
3–5	58 (20.4)	26 (19.4)	32 (21.3)
5–7	35 (12.3)	15 (11.2)	20 (13.3)
> 7	45 (15.8)	20 (14.9)	25 (16.7)
Family history of mental illness			
Yes	36 (12.7)	16 (11.9)	20 (13.3)
No	248 (87.3)	118 (88.1)	130 (86.7)
Comorbid diabetes mellitus			
Yes	14 (4.9)	5 (3.7)	9 (6.0)
No	270 (95.1)	129 (96.3)	141 (94.0)
Duration of DM after onset of CVD illness (year)			
≤ 3	8 (72.7)	3 (75.0)	5 (71.4)
> 3	3 (27.3)	1 (25.0)	2 (28.6)
Comorbid hypertension			
Yes	128 (45.1)	59 (44.0)	69 (46.0)
No	156 (54.9)	75 (56.0)	81 (54.0)
Duration of HTN after onset of CVD illness (years)			
≤ 3	60 (63.8)	27 (62.8)	33 (64.7)
> 3	34 (36.2)	16 (37.2)	18 (35.3)
NYHA class			
I	16 (7.1)	6 (5.8)	10 (8.3)
II	116 (51.8)	54 (52.4)	62 (51.2)
III	57 (25.4)	27 (26.2)	30 (24.8)
IV	35 (15.6)	16 (15.5)	19 (15.7)
Lipid profile level			
Total cholesterol (mg/dl)			
< 200	18 (78.3)	7 (63.6)	11 (91.7)
≥ 200	5 (21.7)	4 (36.4)	1 (8.3)
Low density lipoprotein (mg/dl)			
< 40	9 (36.0)	4 (36.4)	5 (35.7)
≥ 40	16 (64.0)	7 (63.6)	9 (64.3)
Triglycerides (mg/dl)			
< 150	10 (45.5)	4 (40.0)	6 (50.0)
≥ 150	12 (54.5)	6 (60.0)	6 (50.0)

Other diagnosis—corpulmonary, acute rheumatic fever, congenital heart disease, pericarditis, CHF due to thyrotoxicosis

##### New York Heart Association classification among CVD patients and its association with depression

One-half of the participants (51.8%) belonged to NYHA-class II as documented in the secondary data. The prevalence rate of depression in this group was 51.2% (n = 62). This is more than in each of the remaining groups (Table 4).

##### Lipid profile among CVD patients and its association to depression

Cholesterol was investigated and found that patients with a total cholesterol level of < 200 mg/dl constituted in 78% and that they were more depressed 91.7% as to compared with those having total cholesterol level > 200 mg/dl. Those having lipoprotein level > 40 mg/dl were 64% and they were the ones more depressed as compared to those whose LDL level of was < 40 mg/dl. Similarly CVD patients with triglycerides level of < 150 mg/dl were compared with having triglycerides level > 150 mg/dl but there was no statistically significant difference in the level of depression (Table 4).

### Prevalence of depression

**Fig. 1:**
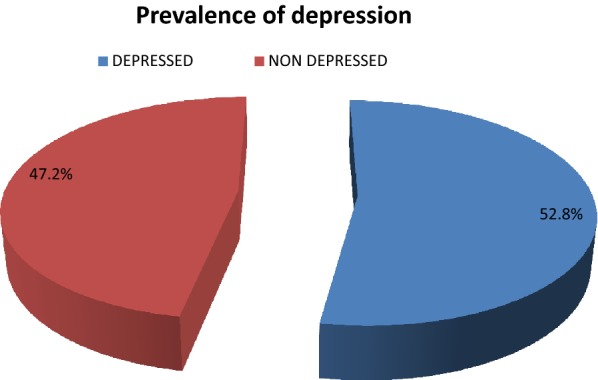
Prevalence of depression among CVD patients in JUTH cardiac clinic, 2014–2015 (n = 284). PHQ-9 classification of Depression in Jimma University Teaching Hospital cardiac clinic, 2014/15 (n = 284)

Two hundred eighty-four of participants completed the interview of PHQ-9 questions at baseline. The prevalence of depression was 52.8% (n = 150) (Fig. 1).

**Fig. 2:**
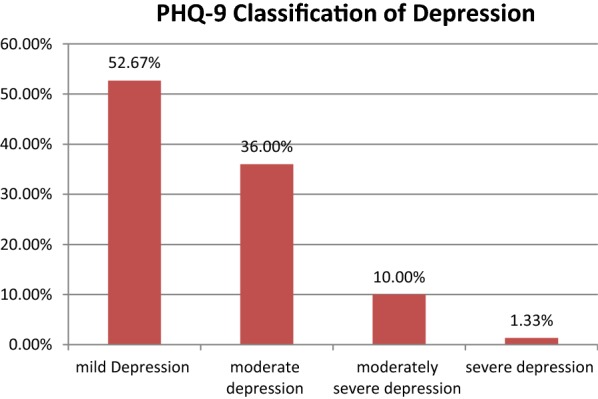
Depression PHQ-9 classification of respondents in Jimma University Teaching Hospital cardiac clinic, 2014/15 (n = 284)

Out of the subjects with depression 52.67% (n = 79), 36.0% (n = 54), 10.0% (n = 15), 1.33% (n = 2) mild, moderate, moderately severe, and severe depression, respectively had depressive symptoms (Fig. 2).

### Factors associated with depression in bivariate analysis among adult CVD patients

Among different variables age, marital status, educational status, employment status, residence, average annual income (according to quartile income classification), living condition, social support, physical activity, current cigarette use, diagnosis type of CVD illness, age onset of CVD, B-blocker medication, duration of treatment for CVD and lipid profile level—total cholesterol were showed association with depression at P-value < .25 on bivariate analysis (Tables 4, 5 and 6).

**Table 5:** Socio-demographic factors that associated with depression in bivariate analysis (< .25) among CVD patients in Jimma University Teaching Hospital cardiac clinic, 2014–2015

Characteristics	Non depressed N (%)	Depressed N (%)	COR (95% CI)	P-value
Age				
18–26	17 (12.7)	17 (11.3)	1.077 (.456, 2.541)	.866
27–35	15 (11.2)	21 (14.0)	1.508 (.644, 3.531)	.344
36–44	20 (14.9)	13 (8.7)	.700 (.291, 1.686)	.426
45–53	22 (16.4)	25 (16.7)	1.224 (.559, 2.678)	.613
54–62	28 (20.9)	26 (17.3)	1	
63–71	22 (16.4)	28 (18.7)	1.371 (.633, 2.968)	.424
≥ 72	10 (7.5)	20 (13.3)	2.154 (.852, 5.448)	.105^a^
Marital status				
Single	20 (14.9)	13 (8.7)	.615 (.291, 1.298)	.202^a^
Married	105 (78.4)	111 (74.0)	1	
Divorced/separate	4 (3.0)	10 (6.7)	2.365 (.720, 7.772)	.156^a^
Widowed	5 (3.7)	16 (10.7)	3.027 (1.071, 8.556)	.037^a^
Educational status				
Illiterate	67 (50.0)	84 (56.0)	1	
Able to read and write only	20 (14.9)	36 (24.0)	1.436 (.762, 2.706)	.263
Primary (1–8)	28 (20.9)	19 (12.7)	.541 (.278, 1.053)	.070^a^
Secondary (9–12)	14 (10.4)	7 (4.7)	.399 (.152, 1.044)	.061^a^
Tertiary (+12)	5 (3.7)	4 (2.7)	.638 (.165, 2.470)	.515
Employment status				
Unemployed	16 (11.9)	35 (23.3)	2.069 (1.053, 4.067)	.035^a^
Employed	12 (9.0)	4 (2.7)	.315 (.097, 1.024)	.055^a^
Farmer	70 (52.2)	74 (49.3)	1	
Merchant	9 (6.7)	10 (6.7)	1.051 (.403, 2.740)	919
Retired	7 (5.2)	5 (3.3)	.676 (.205, 2.228)	.52
Housewife	11 (8.2)	14 (9.3)	1.204 (.512, 2.830)	.67
Other	9 (6.7)	8 (5.3)	.841 (.307, 2.301)	.736
Residence				
Rural areas	80 (59.7)	103 (68.7)	1	
Urban areas	54 (40.3)	47 (31.3)	.676 (.415, 1.101)	.116^a^
Average annual income				
0–999	33 (24.6)	37 (24.7)	1.393 (.723, 2.684)	.322
1000–3599	32 (23.9)	37 (24.7)	1.437 (.743, 2.776)	.281
3600–10,799	28 (20.9)	43 (28.7)	1.908 (.985, 3.694)	.055^a^
≥ 10,800	41 (30.6)	33 (22.0)	1	

^a^Variables which shown statistically significant association during the bivariate analysis

**Table 6:** Psychosocial and behavioral related factors that associated with depression in bivariate analysis (< .25) among CVD patients in Jimma University Teaching Hospital cardiac clinic, 2014–2015

Characteristics	Non depressed N (%)	Depressed N (%)	COR (95% CI)	P-value
Living condition				
Alone	6 (4.5)	12 (8.0)	1.779 (.648, 4.886)	.263
With family	121 (90.3)	136 (90.7)	1	
Other^a^	7 (5.2)	2 (1.3)	.254 (.052, 1.247)	.091^b^
Oslo 3-items social support scale				
Poor support	38 (28.4)	62 (41.3)	1.909 (1.106, 3.293)	.020^b^
Moderate support	62 (46.3)	53 (35.3)	1	
Strong support	34 (25.4)	35 (23.3)	1.204 (.662, 2.189)	.542
Physical activity				
Yes	79 (59.0)	66 (44.0)	.547 (.341, .877)	.012^b^
No	55 (41.0)	84 (56.0)	1	
Cigarette current user				
Yes	3 (2.2)	11 (7.3)	3.456 (.943, 12.664)	.061^b^
No	131 (97.8)	139 (92.7)	1	

Living condition other^a^ with relative/friends/homeless

^b^Variables which shown statistically significant association during the bivariate analysis

Those variables widowed, unemployed, poor social support, physical inactivity was significantly associated with depression in bivariate analysis at P-value < .05 (Tables 5, 6 and 7).

**Table 7:** Clinical and medication related factors that associated with depression in bivariate analysis (< .25) among CVD patients in Jimma University Teaching Hospital cardiac clinic, 2014–2015

Characteristics	Non depressed N (%)	Depressed N (%)	COR (95% CI)	P-value
Diagnosis type of CVD illness				
Hypertensive heart diseases	102 (35.9)	48 (35.8)	1	
Ischemic heart disease	75 (26.4)	37 (27.6)	.913 (.503, 1.658)	.765
Valvular heart disease	35 (12.3)	11 (8.2)	1.939 (.86, 4.371)	.110^a^
Rheumatic heart disease	26 (9.2)	14 (10.4)	.762 (.321, 1.807)	.537
Cardiomyopathy	29 (10.2)	16 (11.9)	.722 (.315, 1.654)	.442
Other	17 (5.0)	8 (6.0)	1.000 (.357, 2.797)	1.00
Age onset of CVD				
9–19	11 (8.2)	12 (8.0)	.679 (.264, 1.745)	.421
20–29	17 (12.7)	21 (14.0)	.769 (.347, 1.701)	.516
30–39	24 (17.9)	19 (12.7)	.493 (229, 1.058)	.070^a^
40–49	24 (17.9)	27 (18.0)	.700 (.339, 1.445)	.335
50–59	30 (22.4)	26 (17.3)	.539 (.266, 1.092)	.086^a^
≥ 60	28 (20.9)	45 (30.0)	1	1
Medication				
B-blocker	72 (53.7)	65 (43.3)	1.519 (.950, 2.426)	.081^a^
Duration of treatment (year)				
≤ 1	91 (32.0)	50 (37.3)	1	
1–2	51 (18.0)	24 (17.9)	1.372 (.69, 2.729)	.367
2–3	29 (10.2)	12 (9.0)	1.728 (.741, 4.028)	.206^a^
3–4	30 (10.6)	11 (8.2)	2.106 (.901, 4.927)	.086^a^
≥ 5	83 (29.2)	37 (27.6)	1.516 (.834, 2.758)	.173^a^
Lipid profile level—total cholesterol (mg/dl)				
< 200	7 (63.6)	11 (91.7)	4.488 (.495, 40.69)	.182^a^
≥ 200	4 (36.4)	1 (8.3)	1	

Diagnosis of CVD other—corpulmonary, acute rheumatic fever, congenital heart disease, pericarditis, CHF due to thyrotoxicosis

^a^Variables which shown statistically significant association during the bivariate analysis

### Factors associated with depression in multiple logistic regression among patients with CVD

Multivariable analysis was done and found that those who were unemployed had about 2 times the odds of developing depression as compared to farmers, adjusted odds ratio (AOR = 2.248, 1.102, 4.583). The odds of developing depression among patients employed were 75% less as compared to farmers (AOR = .252, .073, .869). The odds of developing depression among those who had poor social support was 2 times odd of depression as compared to those with moderate social support (AOR = 2.324, 1.290, 4.187). Similarly, among patients, current cigarette user were 5 times greater odds to develop depression as compared to those who were none current cigarette user (AOR = 4.722, 1.218, 18.311). The odds of developing depression among patients who have a habit of doing physical activity was 53% less as compared to who didn’t do physical activity (AOR = .541, .328, .893) (Table 8).

**Table 8:** Factors that associated with depression in multiple logistic regression of CVD patients in Jimma University Teaching Hospital cardiac clinic, 2014–2015 (n = 284)

Characteristics	Non depression N (%)	Depression N (%)	AOR (95% CI)	P-value
Occupational status				
Unemployed	16 (11.9)	35 (23.3)	2.248 (1.102, 4.583)^a^	.026
Employed	12 (9.0)	4 (2.7)	.252 (.073, .869)^a^	.029
Farmer	70 (52.2)	74 (49.3)	1	
Merchant	9 (6.7)	10 (6.7)	.904 (.326, 2.510)	.847
Retired	7 (5.2)	5 (3.3)	.723 (.212, 2.472)	.605
Housewife	11 (8.2)	14 (9.3)	1.417 (.587, 3.419)	.438
Other	9 (6.7)	8 (5.3)	.635 (.224, 1.805)	.395
Oslo 3-items social support scale				
Poor support	38 (28.4)	62 (41.3)	2.324 (1.290, 4.187)^a^	.005
Moderate support	62 (46.3)	53 (35.3)	1	
Strong support	34 (25.4)	35 (23.3)	1.336 (.702, 2.545)	.378
Current cigarette user				
Yes	3 (2.2)	11 (7.3)	4.722 (1.218, 18.311)^a^	.025
No	131 (97.8)	139 (92.7)	1	
Physical activity				
Yes	79 (59.0)	66 (44.0)	.541 (.328,.893)^a^	.016
No	55 (41.0)	84 (56.0)	1	

^a^Significant level in 95% CI

## Discussion

In this study, an attempt was made to assess the prevalence and associated factors of depression. The prevalence of depression was 52.8% which high as compared to a study done in the US (21.5%), Pakistan (37%), and Jamaica (19.9%) [19, 30, 31]. The prevalence of depression was almost similar to the study done in Washington, USA 51.0% [38]. However, it was lower in magnitude than a study done in Nigerian 67.0% [50], in Brazil 67.0% [32] and USA (Utah) (75%) [27].

In our study, the finding of depression was higher than the studies done in Pakistan (37.0% and 47.0%), Netherlands (42%) Iran (41.9%), Canada (35%) and Nigeria (30.0%) [30, 33–37]. The difference might be studying population, study setting, the tool used, study design and sociocultural difference. The prevalence of depression was higher among CVD patients. This implies that doctors and other professionals should routinely screen depression among patients with CVD.

In this study CVD related employment status, physical activity, current cigarette user and social support were found the potent variable that statically significant association with depression in the final model.

Our study finding revealed that the likelihood of developing depression among those unemployed was 2 times higher than as compared to farmers (AOR = 2.248, 1.102, 4.583). The possibility of developing depression among patients employed was 75% less likely as compared to farmers (AOR = .252, .073, .869). This study in line with the study done in South Korea and the USA [38, 39]. They are more likely to engage in drinking alcohol, smoking, drug use, suicide intentions, and crime. Additionally, because a majority of people among CVD patients were pre-retirement or retirement age was forced to leave their jobs. These things lead to depression among the unemployed [39]. Meta-analyses and systematic reviews did have shown that unemployed have at least twofold risk of mental illness, particularly depression and anxiety disorders, compared to employed persons [40].

In our finding, the likelihood of developing depression among those who had poor social support was 2 times odd of depression as compared to those with moderate social support (AOR = 2.324, 1.290, 4.187). This finding similar to the study done in Canada, Italy, the USA, Brazil, and Iran [41–46]. Low social support levels are important risk factors for the subsequent development or worsening of depression.

In our study finding, among patients who current cigarette user was 5 times more likely to develop depression as compared to those who were none current cigarette user (AOR = 4.722, 1.218, 18.311). This finding similar to the study done in the Netherlands supports that smoking as an independent predictor of depression in CVD patients. Smokers, in turn, have a higher risk of being depressed and a decreased chance of recovery from depression. Both tobacco smoking and depression are associated with cardiac mortality and morbidity [47].

Similarly, the study had done in Australia supports our study finding that reports, smoking as an independent predictor of depression in CVD patients. The mechanisms through which smoking is associated with depression might be biogenetic, psychological and environmental factors. Smoking-induced neurobiological changes that might predispose to depression; the transient alleviation of depressive symptoms and psychotropic side effects with smoking [48]. The study was done in Greece and systematic review done also reported that smokers have higher rates of depressive symptoms than nonsmokers [49, 50]. Smoking cessation among depressed CVD patients is associated with concurrent improvement in depression [51]. Our study finding also contrasts with the study done in Toronto, Canada [52].

In our study participants, those who physically active (do moderate to vigorous activity for 30 min or more, at least 4 days in a week) were 46% less likely to develop depression as compared to physically inactivate, (AOR = .541, .328, .893). This is consistent with the study done in Italy and Spain that report that physical inactivity increases the risk of depression [53, 54]. The study was done in Pakistan also supports our study finding, additionally reported that physical activity shows positive effects to reduce the level of depression among CVD patients [55]. Other studies which were done in Canada and the USA also in line with our study finding [56]. Lastly, in this study respondent’s age, sex, place of residence, B-blocker medication, marital status, comorbid diabetes mellitus and hypertension, living condition and lipid profile were not significantly associated with depression among CVD patients in the final model. The finding was in line with the study done in USA, Netherland, Japan, Denmark, Brazil, Pakistan [12, 20, 27, 30, 32, 38, 53, 57–59].

## Conclusions

Our study finding showed that the prevalence of depression was 52.8% and it was found to be highly prevalent psychiatric comorbidity in adult CVD patients. Depression had independently associated with employment status, physical activity, current cigarette user and social support. This study could be taken as an eye-opener with regards to bridging the knowledge gap that exists in the country and particularly among Jimma University Teaching Hospital’s health workers and specialists working with cardiac patients attending cardiac clinic and could help to put an effort for integration of knowledge from both Internists and psychiatrists through multidisciplinary team approach for achieving quality of patient care.

## Abbreviations

ACS: acute coronary syndrome
AMI: acute myocardial infarction
AF: atrial fiberaltion
BDI: Beck depression inventory
BMI: body mass index
BSC: Bachelor of science
CAD: coronary arterial disease
CABG: coronary artery bypass graft
CHD: coronary heart disease
CHF: congestive heart disease
CVD: cardiovascular disease
CPMJU: College of Public Health Medical Science of Jimma University
DIS: diagnostic interview schedule
DSM-IV: Diagnostic and Statistical Manual of Mental Health Disorders, Fourth Edition
HADS: Hospital anxiety and depression scale
HHD: hypertension heart disease
HRQOL: heart-related quality of life
HTN: hypertension
ICD: implantable cardioverter-defibrillator
IHD: ischemic heart disease
JUST: Jimma University Specialized Hospital
JUTH: Jimma University Teaching Hospital
LVEF: left ventricular ejection fraction
MDD: major depressive disorder
MI: myocardial infarction
NYHA: New York Heart Association
NCDs: non-communicable diseases
PHQ-9: patient health questioner
QOL: quality of life
RHD: rheumatic heart disease
SHHD: Sudan household survey
SPSS: statistical package for social sciences
USA: United States of America
WHO: World health organization
YLL: years of life lost
